# The Synergistic Action of Electro-Fenton and White-Rot Fungi in the Degradation of Lignin

**DOI:** 10.3389/fbioe.2020.00099

**Published:** 2020-03-12

**Authors:** Lipeng Hou, Dandan Ji, Weifang Dong, Lin Yuan, Fengshan Zhang, Yan Li, Lihua Zang

**Affiliations:** ^1^College of Environmental Science and Engineering, Qilu University of Technology, Shandong Academy of Science, Jinan, China; ^2^Huatai Group Corp. Ltd., Dongying, China; ^3^Jiangsu Key Laboratory of Anaerobic Biotechnology, Jiangnan University, Wuxi, China; ^4^Langfang Meihua Biotechnology Development Co. Ltd., Langfang, China

**Keywords:** lignin, electron-Fenton, white-rot fungi, synergistic system, lignin-degrading enzyme

## Abstract

White-rot fungus is a common lignin-degrading fungus. However, compared with those of microorganisms that biodegrade lignin alone, synergistic systems of electro-Fenton processes and white-rot fungi are superior because of their high efficiency, mild conditions, and environmental friendliness. To investigate the details of lignin degradation by a synergistic system comprising electro-Fenton processes and white-rot fungi, lignin degradation was studied at different voltages with three lignin-degrading fungi (*Phanerochaete chrysosporium*, *Lentinula edodes*, and *Trametes versicolor*). The lignin degradation efficiency (82∼89%) of the synergistic systems at 4 V was higher than that of a control at 96 h post inoculation. Furthermore, the H_2_O_2_ produced and phenolic lignin converted in the system can significantly enhance the efficiency of ligninolytic enzymes, so a considerably increased enzyme activity was obtained by the synergistic action of electro-Fenton processes and white-rot fungi. ^13^C NMR spectroscopy revealed that aromatic structure units (103–162 ppm) were effectively degraded by the three fungi. This study shows that the combination of electro-Fenton processes and white-rot fungi treatment significantly improved the lignin degradation efficiency, which established a promising strategy for lignin degradation and valorization.

## Introduction

In the degradation of lignin by microorganisms, synergistic systems have a more significant degradation effect than have systems without synergy. This is due to the non-specific, complex spatial structure of lignin. The lignin structure is characterized by the substitution of methoxycinnamic and hydroxycinnamic acid in the polymerization of heterogeneous 3D crystal polymers and mainly includes three basic components: guaiacyl units (G), syringyl units (S), and *p*-hydroxyphenyl units (H) (Ewellyn et al., 2005; Pinto et al., 2012). These structures are interconnected by C–C single bonds (approximately 30%) and C–O bonds (approximately 60–70%) (Chakar et al., 2004; Pandey and Kim, 2011). Lignin macromolecules contain multiple functional groups, such as hydroxyl, methoxy, carbonyl, and aromatic structures (Azimvand, 2014). High-lignin materials have heterogeneity and structural stability, which are due to the formation and fracture modes of chemical bonds between complex chemical groups, conversions between different chemical groups, and various random interactions. These are the reasons considered challenging for the microbial degradation of aromatic compounds (Wen et al., 2013).

White-rot fungi are among the microorganisms in nature that can mineralize lignin into carbon dioxide and water. Lignin is degraded by their unique H_2_O_2_ production and extracellular enzyme system. The systems involved in lignin degradation mainly include the following. (i) The H_2_O_2_ production system includes glucose oxidase, glyoxal oxidase, and veratryl alcohol oxidase. These enzymes use a small organic molecule as a substrate, and reduction of molecular oxygen to H_2_O_2_ starts the peroxidase reaction process. (ii) The lignin oxidase system involves key enzymes for the degradation of lignin by white-rot fungi, which includes laccase (Lac), manganese peroxidase (MnP), and lignin peroxidase (LiP) (Derrien et al., 2017). MnP and LiP require H_2_O_2_ to trigger their oxidation and activate enzyme cycle reactions. Studies have shown that white-rot fungi are closely connected with the Fenton reaction in the process of lignin degradation. The low-molecular-weight compound, such as organic acids (Galkin et al., 1998), fatty acids (Gutiérrez et al., 2002), Fe^3+^ chelators, and catechol derivatives (Arantes and Milagres, 2006), is secreted by white-rot fungi in the process of wood biodegradation. These substances can reduce Fe^3+^ to Fe^2+^ and generate hydroxyl radicals by the Fenton reaction. The hydroxyl radical is one of the strongest oxidants (E = 2.8 V versus normal hydrogen electrode) and can efficiently and non-selectively oxidize various organic compounds (Cisneros et al., 2002). Hydroxyl radicals can convert non-phenolic lignin into phenolic lignin. Then, MnP and Lac can further oxidize phenolic lignin. This mechanism ensures that lignin can be degraded efficiently when the LiP content is low or absent.

Fe^3+^-reducing compounds and Fenton reagents are commonly used to degrade lignin phenolic resins and enable non-phenolic lignin reactions, which are known as chelator-mediated Fenton reactions (Arantes et al., 2006). White-rot fungi can reduce the pH of the environment as they grow: a substantial reduction in pH was caused by fungi in liquid cultures through metabolic regulation (Jellison et al., 1992; Humar et al., 2001). This change is attributed to fungi that can secrete and produce many acidic group-containing compounds, such as phenolic acids and other simple organic acids, during metabolism (Jellison et al., 1997).

There are limitations of single-component biodegradation, such as lone reaction time and high environmental requirements, and this study attempted to adopt a synergistic system of biodegradation and electro-Fenton. Electro-Fenton technology is a new technology of lignin treatment. It is a combination of Fenton oxidation and electrochemical technology, which has unique advantages in the treatment of refractory organic pollutants (Pulgarin and Kiwi, 1996). Composite cathode electro-Fenton (CCEF) technology is an improved electro-Fenton technology that can produce H_2_O_2_ and Fenton reagents *in situ* without the exogenous addition of Fe^2+^. Dioxygen in solution is reduced by two electrons at the cathode and protonated to H_2_O_2_, which then reacts with Fe^2+^ to produce hydroxyl radicals while Fe^2+^ is oxidized to Fe^3+^. The newly generated Fe^3+^ can be regenerated on the surface of the electrode to improve the efficiency of the iron catalyst. The cathode reactions are as follows (Eqs 1–3) (Kurt et al., 2007; Ting et al., 2008):

(1) $${\text{O}}_{2}+2\,{\text{H}}^{+}+2\,{\text{e}}^{-}\to {\text{H}}_{2}\,{\text{O}}_{2}$$

(2) $${\text{H}}_{2}{\text{O}}_{2}+{\text{Fe}}^{2+}\to ∙\text{OH}+{\text{OH}}^{-}+{\text{Fe}}^{3+}$$

(3) RH+∙OH→Products

Fe^3+^ can be reduced to Fe^2+^ by reducing substances in the solution or on the cathode surface (Eqs 4–6):

(4) $${\text{Fe}}^{3+}+{\text{H}}_{2}{\text{O}}_{2}\to {\text{Fe}}^{2+}+{\text{HO}}_{2}∙+{\text{H}}^{+}$$

(5) $${\text{Fe}}^{3+}{\text{HO}}_{2}∙\to {\text{Fe}}^{2+}+{\text{O}}_{2}+{\text{H}}^{+}$$

(6) $${\text{Fe}}^{3+}+{\text{e}}^{-}\to {\text{Fe}}^{2+}$$

The following side reactions are present in the solution (Eqs 7–12):

(7) $${\text{Fe}}^{2+}+∙\text{OH}\to {\text{Fe}}^{3+}+{\text{OH}}^{-}$$

(8) $${\text{H}}_{2}{\text{O}}_{2}+∙\text{OH}\to {\text{HO}}_{2}∙+{\text{H}}_{2}\text{O}$$

(9) $${\text{Fe}}^{2+}+{\text{HO}}_{2}∙+{\text{H}}^{+}\to {\text{Fe}}^{3+}+{\text{H}}_{2}{\text{O}}_{2}$$

(10) $$∙\text{OH}+{\text{HO}}_{2}∙\to {\text{H}}_{2}\text{O}+{\text{O}}_{2}$$

(11) $$2{\text{HO}}_{2}∙\to {\text{H}}_{2}{\text{O}}_{2}+{\text{O}}_{2}$$

(12) $$2∙\text{OH}\to {\text{H}}_{2}\,{\text{O}}_{2}$$

This experiment aimed to study the effect of lignin decomposition in a constructed synergistic system. The synergistic system is a lignin fungus culture containing a composite cathode loaded with Fe^0^ to which a voltage has been applied. The growth of fungi decreased the pH of the environment and provided acidic conditions for the Fenton reaction. The Fe^0^ becomes Fe^2+^ in the composite cathode under an applied potential. At the same time, there was a continuous Fenton reaction between Fe^2+^ and H_2_O_2_ produced by the composite cathode, and the large number of hydroxyl radicals produced degraded lignin. H_2_O_2_ can also quickly start LiP and MnP enzymatic hydrolysis reactions to accelerate lignin biodegradation (Mir-Tutusaus et al., 2018). In addition, the voltage promoted the growth of fungal cells (Thrash and Coates, 2008; Loghavi et al., 2010b), so that more lignin-degrading enzymes were produced. The effect of lignin degradation in this mutually advantageous synergistic system was also investigated. This is the main content of this paper.

## Materials and Methods

### Materials

Dealkaline lignin, a mixture of different herbaceous plants, such as corn stover, bamboo, and straw, was obtained from Shanghai Chemical Reagent Four Factory (Shanghai, China).

The white-rot fungi medium included dealkaline lignin (500 mg/L), KH_2_PO_4_ (2 g/L), MgSO_4_ (0.25 g/L), CaCl_2_ (0.1 g/L), MnSO_4_ (5 mg/L), VB_1_ (10 mg/L), ammonium tartrate (0.2 g/L), and trace element solution (150 ml/L). The trace element solution included NaCl (1.0 g/L), FeSO_4_⋅7H_2_O (100 mg/L), CoSO_4_⋅7H_2_O (100 mg/L), CaCl_2_ (100 mg/L), ZnSO_4_⋅7H_2_O (100 mg/L), CuSO_4_⋅5H_2_O (10 mg/L), KAl(SO_4_)_2_ (100 mg/L), H_3_BO_3_ (10 mg/L), and Na_2_MoO_4_ (10 mg/L). All reagents were purchased from Sinopharm Chemical Reagent Co., Ltd., (Beijing, China).

Fabrication of composite electrodes [Fe^0^ and Fe_3_O_4_/activated carbon fiber (ACF)]: An ACF (3 × 10 cm) was ultrasonically cleaned in water for 10 min and then dried in a blast oven. The pretreated ACF was soaked in 100 ml of solution for 1 h. The solution was an 80% ethanol aqueous solution in which 9.66 g of FeCl_3_⋅6H_2_O was dissolved. NaBH (3.54 g) was preliminarily dissolved in 100 ml of deionized water, and the NaBH_4_ solution was dropwise added to the ACFs in a surface pan at a speed of 0.5 ml/s using a dropper. The whole operation was completed at room temperature. During the drip process, a large number of bubbles were produced, and black fluffy substances were generated on the ACFs. After the completion of 1 h of dropwise addition, residual NaBH_4_ on the carbon fiber surface was removed with deionized water and ethanol. Under the protection of argon, infrared light drying was performed, and composite cathodes (Fe^0^ and Fe_3_O_4_/ACF) were obtained.

A constant current was supplied using a DC power supply (Zhaoxin, China, 0–3 A, 0–5 V).

### Fungal Strains and Inoculation

*Phanerochaete chrysosporium* (CICC14076), *Lentinula edodes* (CICC14019), and *Trametes versicolor* (CICC50001) were purchased from the China Industrial Bacteria Conservation Center.

These strains were preserved on potato dextrose agar (PDA) plates at 4°C. They were inoculated into the white-rot fungi medium with an inoculation loop (1 μl) and incubated for 4 days at 39°C.

### Analytical Methods

#### Lignin Removal Efficiency Determination

The sample was first hydrolyzed for 30 min at room temperature using 72% (w/w) H_2_SO_4_. Then it was hydrolyzed a second time for 60 min at 120°C with 4% H_2_SO_4_. The solid residue obtained after acid hydrolysis was determined. Ash content was determined in an oven at 550°C over 8 h. Lignin content on free ash basis is the difference between the solid residue and ash (Ballesteros et al., 2004).

The lignin degrading ratio was calculated using the formula *R* (%) = 100 × (*m*_0_-*m*)/*m*_0_, where *R* is the degrading ratio for the sample; *m*_0_ is the initial content of lignin; *m* is the sampling content of lignin.

#### Ligninolytic Enzyme Activity

The enzyme activity was determined in 5 g of culture that was suspended in 100 ml of sodium acetate buffer (1 mM) and pH 5 and vigorously blended for 1 min in a Waring blender. The Lac activity was tested in 1 mM of sodium acetate buffer at pH 5 with 0.5 M of 2,2′-azinobis-(3-ethylbenzothiazoline-6-sulfonate) (ABTS) (Bourbonnais et al., 1995) and measured in a microplate reader at 420 nm (ε_420_, 36,000 M^–1^ cm^–1^) with a distance of 0.29 cm. The LiP activity was tested in 125 mM of sodium tartrate at pH 3 using 2 mM of hydrogen peroxidase and 0.16 mM of azure B (Archibald, 1992) and measured in a microplate reader at 610 nm (ε_651_, 48,800 M^–1^ cm^–1^). The MnP activity was determined in 50 mM of sodium succinate (pH 4.5) and 50 mM of sodium lactate (pH 4.5) using 0.1 mM of MnSO_4_, 0.1 mM of phenol red, and 50 μM of H_2_O_2_ and measured in a microplate reader at 610 nm (ε_651_, 30,737 M^–1^ cm^–1^).

#### ^13^C NMR

Nuclear magnetic resonance (NMR) spectra of lignin media were acquired at 298 K on an AVANCE III HD 500 MHz instrument (Bruker, Switzerland). The sample (80 mg) was dissolved in 0.5 ml of DMSO-*d*_6_ (99.8%). The parameters were as follows: pulse angle (30°), pulse width (9.2 μs), delay time (1.00 s), and acquired time (3.28 s).

#### Expression of Lac and Versatile Peroxidase Transcripts by Real-Time Polymerase Chain Reaction

The total RNA of *P. chrysosporium* and *L. edodes* mycelia growing in different electro-Fenton levels using lignin medium was isolated by TRIzol reagent (Invitrogen, Carlsbad, CA, United States). The RNA concentration was determined by absorbance at 260 nm. Subsequently, 2 μg of RNA was reverse-transcribed in a 50 μl reaction mixture using the iScript^TM^ complementary DNA (cDNA) synthesis kit (Bio-Rad Laboratories, Hercules, CA, United States). The cDNA samples were stored at −20°C for use.

To determine the mRNA levels of the *LiP*, *MnP*, and *Lac* genes, quantitative real-time PCR was performed with a CFX96 real-time system (Bio-Rad Laboratories, Hercules, CA, United States) and SYBR green I in 96-well plates. The volume of each PCR sample was 20 μl, which included 0.5 μl of PCR reverse primer, 10 μl of SYBR green I, 0.5 μl of PCR forward primer, 5 μl of cDNA, and 4 μl of distilled water (DW). The β-actin gene of *P. chrysosporium* was used as an internal control. The β*-tubulin* gene of *L. edodes* was used as an internal control. The primer pairs for intergenic regions are shown in Table 1.

**TABLE 1:** Primer pairs of the real-time PCR.

Strain	Gene	Primer pairs
*P. chrysosporium*	*LiP*	F: 5′-TTCTTCGTCGAGACTCAG-3′
		R: 5′-CTTGGACTGGTTGTTGAC-3′
*P. chrysosporium*	*MnP*	F: 5′-CTGTGAGTTACGGAATTGG-3′
		R: 5′-GGAGGAGGAGGAAGTAGA-3′
*P. chrysosporium*	*β-Actin*	F: 5′-GCACCACACCTTCTACAA-3′
		R: 5′-TCATCTTCTCACGGTTAGC-3′
*L. edodes*	*LiP*	F: 5′-GACGCCTTTCCCTGCTCTTACTG-3′
		R: 5′-AGCAGCAGAACGAGCATATTCAGG-3′
*L. edodes*	*MnP*	F: 5′-CAATCCTCCGCTGTTACA-3′
		R: 5′-TCGTTCGTGACCAAGATAA-3′
*L. edodes*	*Lac*	F: 5′-CCAACCATTCGTTCCTCTA-3′
		R: 5′-GCAGTCAGCAATAGTAAGC-3′
*L. edodes*	*β-Tublin*	F: 5′-TTCACCGACAACATCACT-3′
		R: 5′-GACATAACAAGGGACACAAG-3′
*T. versicolor*	*LiP*	F: 5′-CACGACCTTTCCATCTTC-3′
		R: 5′-ATGCTTGCTGGTTAGTTG-3′
*T. versicolor*	*MnP*	F: 5′-GACACGCAGTTCTTCATC-3′
		R: 5′-CTGGTTGTTGACGAAGGA-3′
*T. versicolor*	*Lac*	F: 5′-GCTATCCTCCGCTATGAT-3′
		R: 5′-GCCGTTGATGAAGAAGTT-3′
*T. versicolor*	*β-Actin*	F: 5′-TATCCGTCGTGACCTCTA-3′
		R: 5′-CGATCTTGACCTTCATACTTG-3′

*All data were normalized to the internal control, and relative expression was calculated using the 2-DDCt method. The results were analyzed by one-way analysis of variance (ANOVA). Statistical analysis was performed using Statistical Product and Service Solutions (SPSS).*

## Results and Discussion

### Determination of Optimal Voltage for Electro-Fenton Reaction

**FIGURE 1:**
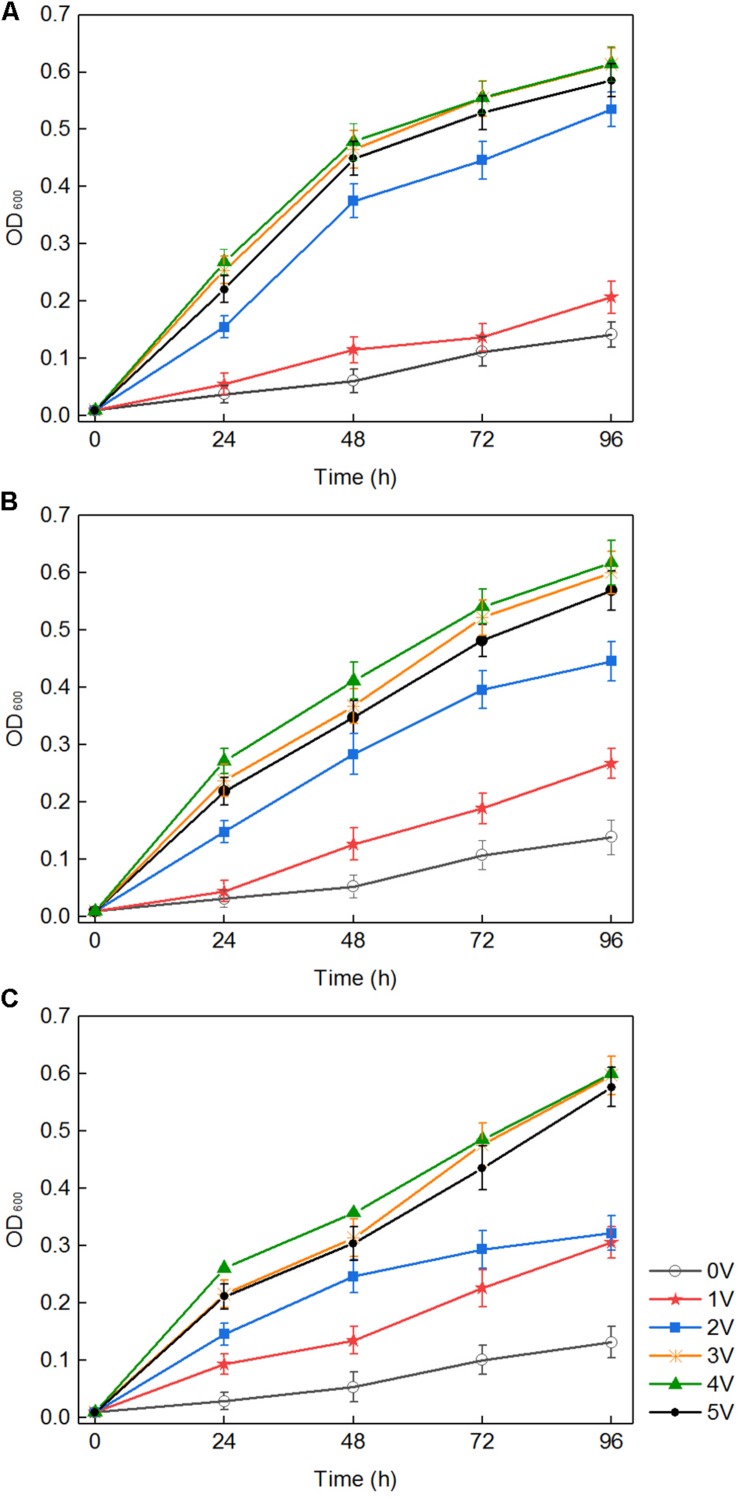
Growth curves of three fungi treated with different voltages: *Phanerochaete chrysosporium* **(A)**, *Trametes versicolor* **(B)**, and *Lentinula edodes* **(C)**.

In a certain range, the applied voltage clearly promoted the growth of the three white-rot fungi (*P. chrysosporium*, *T. versicolor*, and *L. edodes*). As shown in Figure 1, in the absence of an applied voltage in the control, white-rot fungi grew slowly, and the highest optical density (OD) of *P. chrysosporium*, *T. versicolor*, and *L. edodes* was only 0.142, 0.132, and 0.139 within 96 h, respectively. However, under an applied voltage, the growth of the three fungi rapidly increased with increasing voltage. The results also showed that different voltages had different effects on the three white-rot fungi; and 1, 2, 3, and 4 V promoted the growth of the three white-rot fungi to varying degrees. The higher the voltage was in the range of 1–4 V, the more obvious the promoting effect. The OD of *P. chrysosporium*, *T. versicolor*, and *L. edodes* reached the highest values (0.615, 0.618, and 0.601, respectively) at 4 V. However, the OD slightly decreased at 5 V, suggesting that 4 V is the best voltage for the growth of these three white-rot fungi.

**FIGURE 2:**
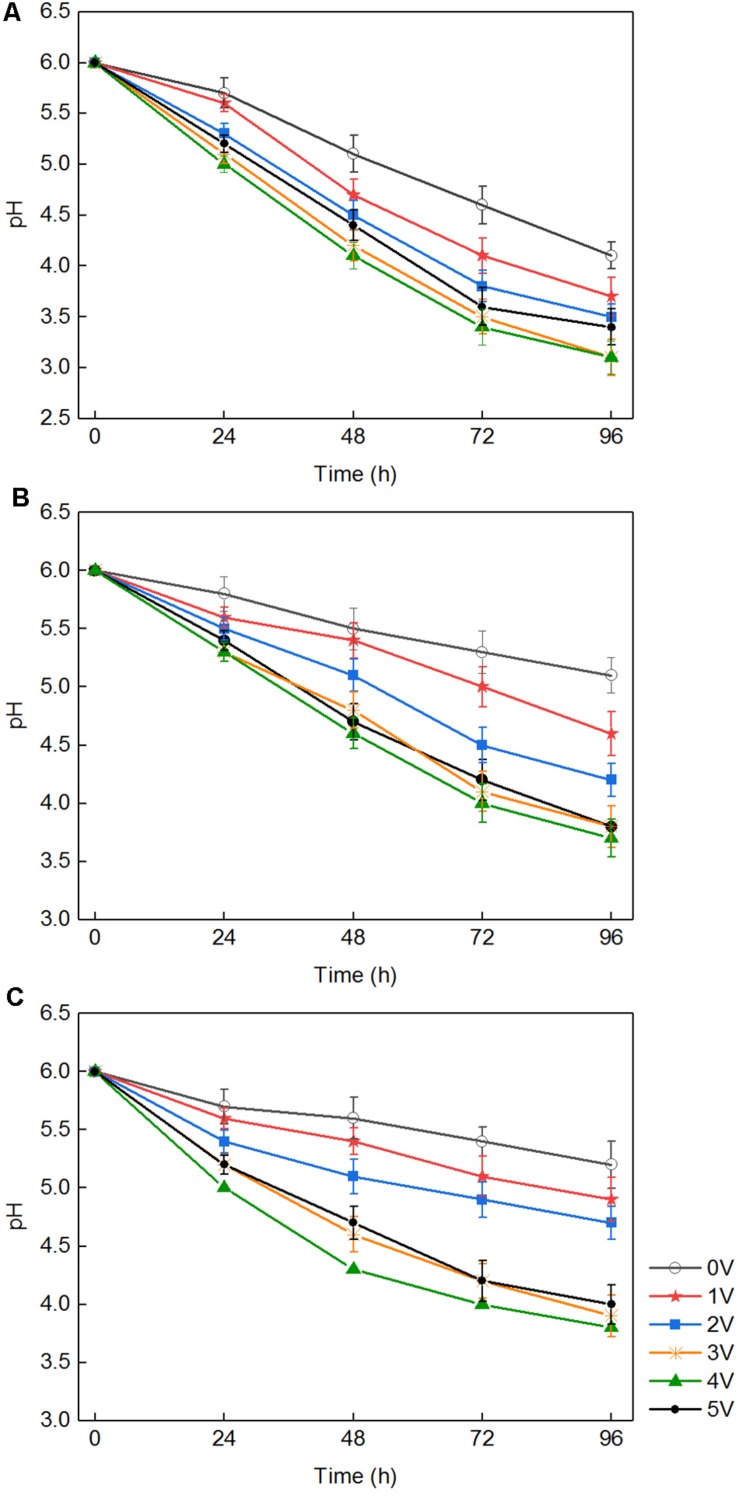
The pH of the medium after being cultured with three fungi with different voltages: *Phanerochaete chrysosporium* **(A)**, *Trametes versicolor* **(B)**, and *Lentinula edodes* **(C)**.

There were three ways to explain the above phenomenon. First, in the medium with white-rot fungi, the pH dropped rapidly to approximately 3.5 (Figure 2). H_2_O_2_ and Fe^2+^ generated in the composite cathode under an applied voltage reacted to produce hydroxyl radicals, which occurred via the Fenton reaction. The macromolecular groups were converted into small molecules that could be easily used by white-rot fungi (Kai et al., 2018), which was conducive to the transport of nutrients into the cell and promoted the growth and reproduction of white-rot fungi. Second, it was reported that an external voltage can change the cell membrane permeabilization (Loghavi et al., 2010a), which indicates that the permeability of the fungal cell membrane could be improved by application of an appropriate voltage. For cells grown in liquid medium, because of the diffusion of nutrients in the liquid matrix, the increased membrane permeability promoted the diffusion of nutrients on cell membranes. Third, an applied voltage increased intracellular protein and ATP levels and promoted the metabolic ability of cells to a certain extent (Filipič et al., 2012). Previous studies suggested that temporary, non-lethal membrane permeabilization occurs when cells are subjected to suitable voltage, which is conducive to introducing various exogenous substances into living cells. However, because of the permeability of cell membrane, the growth and metabolism of white-rot fungi could be inhibited when the applied voltage was over the capacity of microorganism (Loghavi et al., 2010c). This is the reason for the slow growth of white-rot fungi at 5 V.

### The Improvement of Lignin Degradation by Electro-Fenton Reaction Analysis of Lignin Degradation Efficiency

In the medium containing lignin, *P. chrysosporium*, *T. versicolor*, and *L. edodes* were cultured for 96 h. As shown in Table 2, in the absence of voltage, the degradation rates of lignin for *P. chrysosporium*, *T. versicolor*, and *L. edodes* were 17, 19, and 19%, respectively. The degradation rate of lignin at 4 V was approximately 19% when there was no white-rot fungus. In the presence of white-rot fungi and an applied voltage, the degradation rates of lignin for *P. chrysosporium*, *T. versicolor*, and *L. edodes* at 4 V were 82, 86, and 89%, respectively, which were 65–70% higher than those obtained with white-rot fungi or applied voltage alone.

**TABLE 2:** Lignin degradation efficiency by three fungi at different voltages.

Strains	Lignin degradation efficiency (%) at different voltages					
	
	0V	1V	2V	3V	4V	5V
*P. chrysosporium*	17 ± 1	23 ± 2	50 ± 2	79 ± 3	82 ± 3	78 ± 2
*T. versicolor*	19 ± 2	28 ± 3	55 ± 2	77 ± 3	86 ± 3	75 ± 2
*L. edodes*	19 ± 2	34 ± 3	60 ± 3	87 ± 4	89 ± 3	85 ± 3
No fungi	N/A	7 ± 1	12 ± 2	15 ± 1	19 ± 1	22 ± 2

**FIGURE 3:**
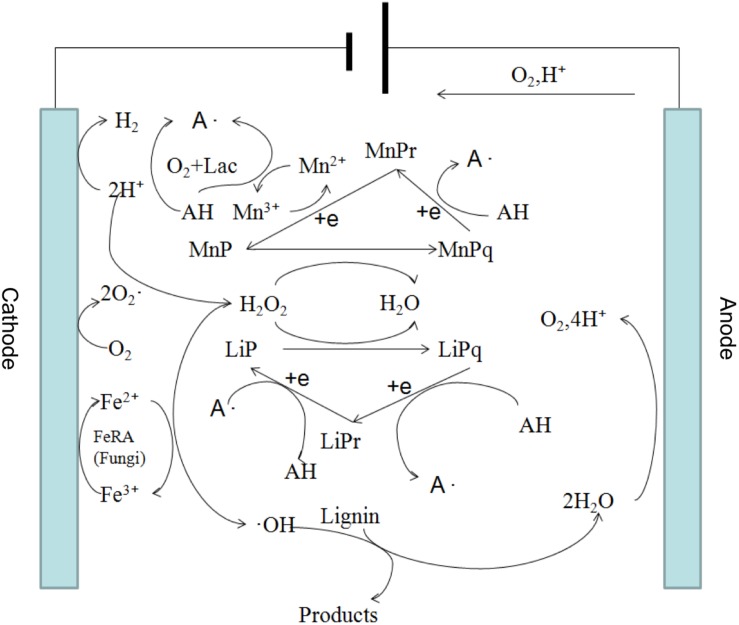
Proposed mechanism of the synergistic degradation of lignin by electro-Fenton reaction and white-rot fungi.

These results indicate that a synergistic effect on lignin degradation occurred in the presence of white-rot fungi and applied voltage. The reasons for the synergistic effect can be summarized as follows. First, under an appropriate voltage for the electro-Fenton reaction, the pH of the liquid medium decreased rapidly owing to the growth of white-rot fungi (Figure 2) and reached the appropriate acidity required by the Fenton reaction. The Fenton reaction occurred between the H_2_O_2_ and the Fe^2+^ generated under the applied voltage on the composite cathode. Large numbers of hydroxyl radical (OH) and various functional groups in lignin were degraded by direct oxidation. The degradation of lignin was promoted. Second, in the presence of an applied voltage, H_2_O_2_ produced by the composite cathode initiated the enzymatic hydrolysis reactions of LiP and MnP secreted by white-rot fungi (Mir-Tutusaus et al., 2018), thus accelerating the lignin biodegradation. The mechanism is shown in Figure 3. Third, the hydroxyl radical produced by the Fenton reaction and LiP synergized to convert non-phenolic lignin into phenolic lignin (Ohashi et al., 2011), which further improved the ability of MnP and Lac to degrade lignin.

#### The Structure Analysis of Lignin During the Process of Degradation

**FIGURE 4:**
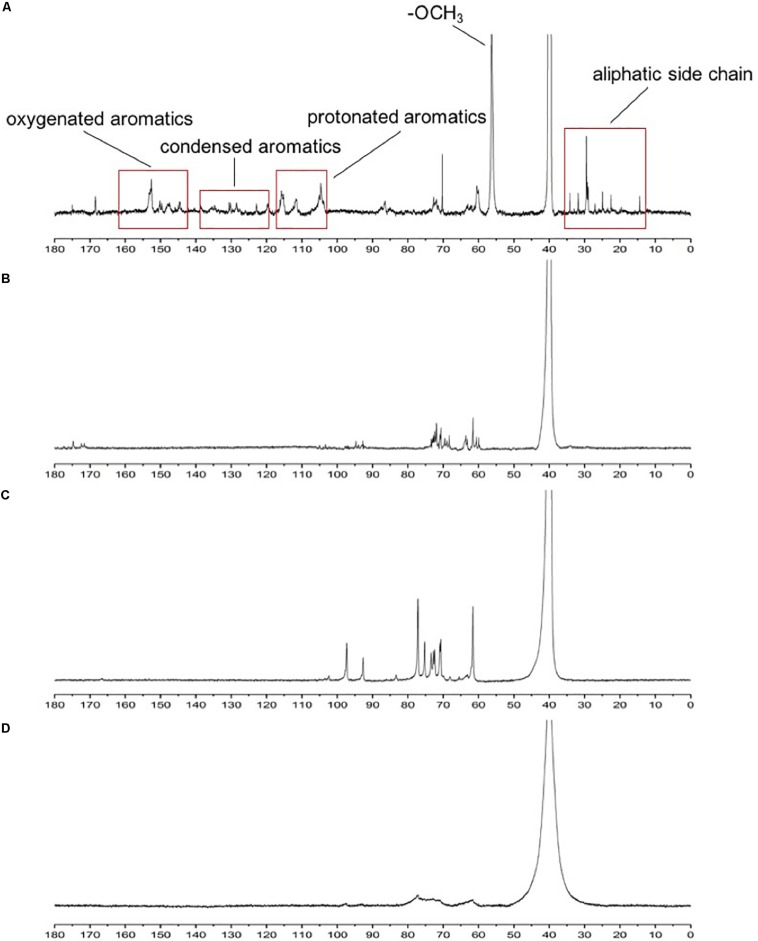
^13^C NMR spectra of standard lignin sample **(A)** and lignin samples after being cultured with *Phanerochaete chrysosporium* **(B)**, *Trametes versicolor* **(C)**, or *Lentinula edodes* **(D)**.

^13^C NMR is often used to determine the structural changes in lignin during biodegradation. ^13^C NMR spectra of lignin were analyzed before and after degradation. In the ^13^C NMR spectra (Figure 4), the aromatic region (103–162 ppm) of undegraded lignin (Figure 4A) was easily identified by the correlated signals at 104.2, 111.1, 115.6, 119, 128, 130.1, 147.0, 150, and 152.3 ppm, corresponding to the S_2_,_6_, G_2_, G_5_, G_5_,_6_, H_2_,_6_, pCA_2_,_6_, G_4_, G_3_, and S_3_,_5_ positions, respectively, as shown in Table 3 (Capanema et al., 2004; Sun et al., 2016; Ying et al., 2018). The signal intensities of the aromatic region (based on the signals at 103–162 ppm) (Ying et al., 2018) in the degraded lignin decreased significantly or even disappeared after degradation by the three white-rot fungi. The changes in the above signals indicated that degradation by *P. chrysosporium*, *L. edodes*, and *T. versicolor* can reduce the aromatic structures in lignin.

**TABLE 3:** Signal assignments of lignin medium samples in the ^13^C NMR.

δ (ppm)	Assignments	δ (ppm)	Assignments
152.3	C_3_/C_5_ in S unit	115.6	C_5_ in S unit
150–148	C_3_ in G unit	111.1	C_2_ in G unit
147.0	C_4_ in G unit	104.2	C_2_/C_6_ in S unit
130.1	C_2_/C_6_ in pCA unit	62–75	Interunit linkages
128	C_2_/C_6_ in H unit		(β-5, β-β and β-O-4)
119	C_6_/C_5_ in G unit	56.5	OCH_3_ in G and S unit

*G, guaiacyl unit; S, syringyl unit; H, *p*-hydroxyphenyl unit; pCA, esterfied *p*-coumaric acid.*

Moreover, decreasing signal intensities for –OCH_3_ were observed at 56.5 ppm, implying that the degradation of lignin by the three studied white-rot fungi could cause demethoxylation and demethylation. The bands of β-5, β-β, and β-O-4 (62–75 ppm) (Evstigneyev et al., 2018) became weak and narrow in Figure 4D compared with those in Figures 4B,C, implying that *L. edodes* had a more significant influence on lignin during the degradation than the other two fungi (Sun et al., 2016).

### Improvement of Lignin-Degrading Enzyme Activity by Electro-Fenton Reaction

#### Analysis of Lignin-Degrading Enzyme Activity

**FIGURE 5:**
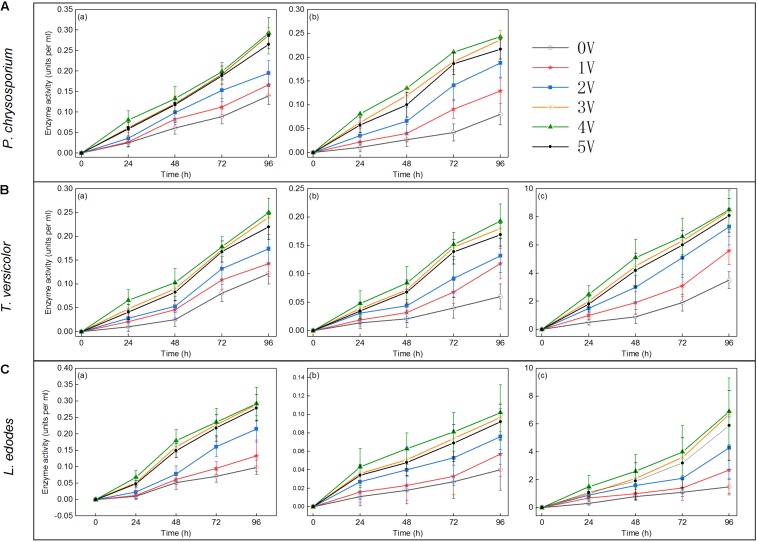
Enzyme activities (units per milliliter) in lignin samples after treated with *Phanerochaete chrysosporium* **(A)**, *Trametes versicolor* **(B)**, or *Lentinula edodes* **(C)** at the 24th, 48th, 72nd, and 96th hours post inoculation: MnP **(a)**, LiP **(b)**, and Lac **(c)**.

**FIGURE 6:**
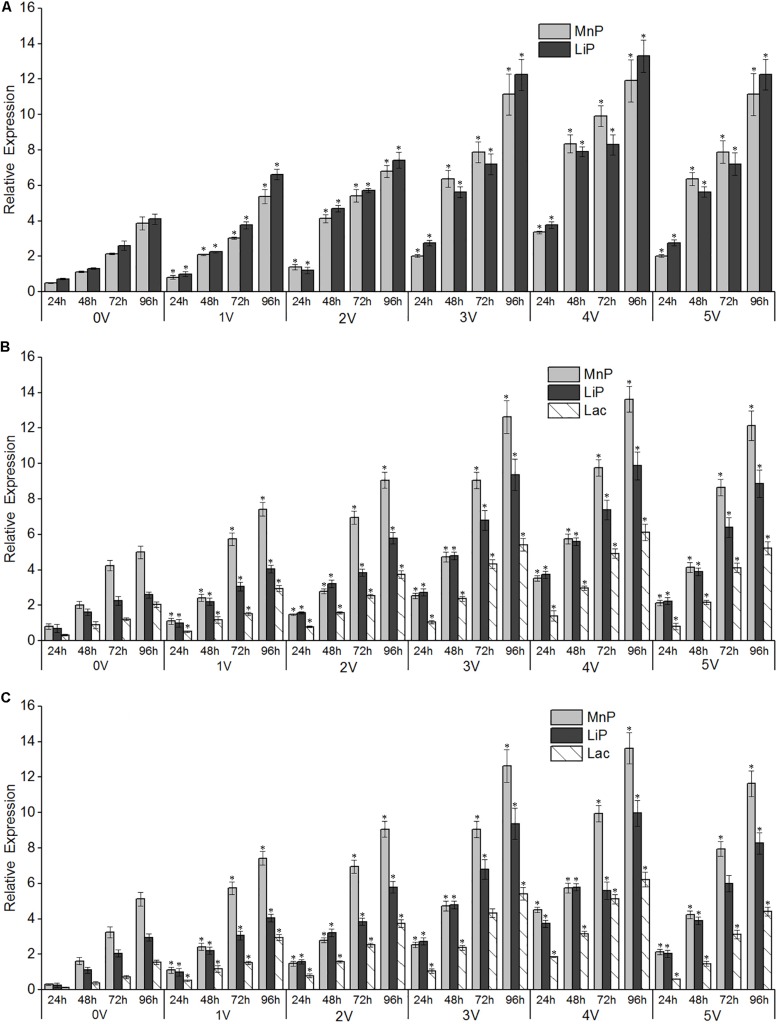
Effects of different electro-Fenton levels on the mRNA expression patterns of lignin-degrading enzymes in *Phanerochaete chrysosporium* **(A)**, *Trametes versicolor* **(B)**, and *Lentinula edodes* **(C)**. Quantitative real-time PCR analysis was performed with samples obtained after 96 h of cultivation of white-rot fungi at varying electro-Fenton concentrations. Asterisk indicates significance at *p* < 0.05. β-Actin **(A,C)** and β-tubulin **(B)** were used as internal control genes for normalization.

Lignin hydrolase is a peroxidase that oxidizes lignin during the depolymerization of lignin structure (Tekere et al., 2001). Similar to previous results (Maria et al., 2009), the data showed that significant lignin degradation always accompanied an increase in a lignin-degrading enzyme activity. Lignin degradation was achieved by the synergistic action of various enzymes, and the applied voltage significantly affected the activity of lignin-degrading enzymes. As shown in Figure 5, MnP, LiP, and Lac activities increased with increasing voltage within 96 h. The main reason for the above phenomenon was that the biomass of white-rot fungi and the expression of enzyme gene were significantly increased under voltage stimulation (Figures 1, 6). Lac activity of *P. chrysosporium* was not detected in this study (Figure 5A), which was similar to the results of a previous study (Dong et al., 2013). The activities of three enzymes (MnP, LiP, and Lac) in *T. versicolor* were slightly lower than those in *P. chrysosporium* (Figure 5B); that is, the MnP activity was relatively small and the LiP growth rate was low. However, among the three enzymes measured for *L. edodes* (Figure 5C), both LiP and Lac had low enzyme activity, and the Lac activity increased rapidly at the 72nd hour. These results differ from those of a previous study (Osma et al., 2011), which revealed that Lac activity remained consistent over time. In the current study, increases in LiP activity may be affected by the color of aromatic compounds or the presence of some inhibitors of veratryl alcohol oxidation, as reported in another study (Maria et al., 2009). Overall, the enzyme activity increased with increasing voltage, and the effect of voltage on a lignin-degrading enzyme activity was significant.

#### Enzyme Gene Expression

There was a significant correlation between enzyme activity and gene expression, as shown in Figure 6, and the relative expression of LiP genes in the three strains increased with increasing treatment time. The voltage had an obvious promoting effect on the expression of *LiP* genes, and the expression of *LiP* genes increased with increasing voltage. In *P. chrysosporium*, both *LiP* and *MnP* gene expression was induced by electro-Fenton treatment, and the highest expression was observed at the 96th hour at 4 V. In *L. edodes* and *T. versicolor*, the gene expression of *LiP*, *MnP*, and *Lac* was induced by electro-Fenton treatment, and the highest expression was observed at the 96th hour with an applied voltage of 4 V. In *P. chrysosporium* and *L. edodes*, the expression of the *LiP* gene was the highest, whereas the expression of the *MnP* gene was the highest in *T. versicolor*.

Lacs are involved in functions such as host–pathogen interactions, stress defenses, and the degradation of lignin and many xenobiotic compounds. Such ligninolytic enzymes are widely used in biotechnology. Therefore, the regulation of *Lac*, *LiP*, and *MnP* gene expression and the activity of enzymes in the white-rot fungi, *P. chrysosporium*, *T. versicolor*, and *L. edodes*, grown in liquid culture for 96 h with different levels of electro-Fenton treatment, were studied. The result of increased Lac activity at low levels of electro-Fenton treatment was consistent with results of a previous report that showed the stimulatory effects of electro-Fenton treatment on the Lac activity of *P. chrysosporium* and *L. edodes* (Merve and Raziye Ozturk, 2014). The stimulatory effects of electro-Fenton treatment on LiP and MnP activity in *P. chrysosporium* have also been noted (Couto et al., 2005).

In the presence of low concentrations of OH^–^, incubation of fungal cultures also led to the depletion of peroxidase (Welinder, 1992). In contrast, lignin-degrading enzyme transcripts were found at high levels in mycelium growth medium receiving electro-Fenton treatments. This result was probably related to the improved antioxidant status of the organism because of the induction of proteins in a stressed reactive oxygen species (ROS) environment, probably through a stress response mechanism. This study showed the highest expression of ligninolytic enzyme genes in all three white-rot fungi. Similar studies have been conducted on the regulation of peroxidases and especially oxidative stress with peptone-containing liquid cultures of *Pleurotus eryngii*. In these cases, the maximal peroxidase messenger RNA (mRNA) content was detected 15 min after the initiation of stress, suggesting a rapid response (Ruiz et al., 2010).

## Conclusion

Electro-Fenton processes promoted the degradation of lignin by white-rot fungi. For the three white-rot fungi, the lignin degradation rate increased with increased voltage of the electro-Fenton processes within a certain range. The highest degradation rates were 82% for *P. chrysosporium*, 86% for *T. versicolor*, and 89% for *L. edodes*, which were achieved at 4 V. Aromatic structures (103–162 ppm) in lignin were degraded, and *L. edodes* under electro-Fenton treatment exhibited significant delignification activity. The biological characteristics of the three white-rot fungi changed significantly compared with those of the control. Owing to the applied voltage and the accompanying production of H_2_O_2_ by electro-Fenton processes, the OD, ligninolytic enzyme activity, and the relative expression of ligninolytic enzyme genes were greatly improved. Overall, electro-Fenton processes increased the activity of ligninolytic enzymes and had a synergistic effect with white-rot fungi on lignin degradation.

## Data Availability Statement

All datasets generated for this study are included in the article/supplementary material.

## Author Contributions

LH and LZ designed the research. LZ guided the writing, LH, DJ, and LY performed the research. LH, DJ, WD, and LZ analyzed the data. FZ and LY contributed new reagents and analytic tools. LH and WD wrote the manuscript. All authors have read and approved the final manuscript.

## Conflict of Interest

DJ and FZ were employed by company Huatai Group Corp. Ltd. YL was employed by company Langfang Meihua Biotechnology Development Co. Ltd. The remaining authors declare that the research was conducted in the absence of any commercial or financial relationships that could be construed as a potential conflict of interest.

## Abbreviations

ABTS: 2,2′-azinobis-(3-ethylbenzothiazoline-6-sulfonate)
ACF: activated carbon fiber
ANOVA: analysis of variance
CCEF: composite cathode electro-Fenton
cDNA: complementary DNA
DC: direct current
H_2_O_2_: hydrogen peroxide
Lac: laccase
LiP: lignin peroxidase
MnP: manganese peroxidase
mRNA: messenger RNA
NMR: nuclear magnetic resonance
OD: optical density
PDA: potato dextrose agar
PPO: polyphenol oxidase
real-time PCR: real-time polymerase chain reaction
ROS: reactive oxygen species
SPSS: Statistical Product and Service Solutions.
